# Genetically proxied therapeutic prolyl-hydroxylase inhibition and cardiovascular risk

**DOI:** 10.1093/hmg/ddac215

**Published:** 2022-09-01

**Authors:** Charli E Harlow, Vickas V Patel, Dawn M Waterworth, Andrew R Wood, Robin N Beaumont, Katherine S Ruth, Jessica Tyrrell, Asami Oguro-Ando, Audrey Y Chu, Timothy M Frayling

**Affiliations:** College of Medicine and Health, University of Exeter, Exeter, Devon EX2 5DW, UK; GlaxoSmithKline, Collegeville, PA 19426, USA; Spark Therapeutics, Inc., Philadelphia, PA 19104, USA; GlaxoSmithKline, Collegeville, PA 19426, USA; Immunology Translational Sciences, Janssen, Spring House, PA 19044, USA; College of Medicine and Health, University of Exeter, Exeter, Devon EX2 5DW, UK; College of Medicine and Health, University of Exeter, Exeter, Devon EX2 5DW, UK; College of Medicine and Health, University of Exeter, Exeter, Devon EX2 5DW, UK; College of Medicine and Health, University of Exeter, Exeter, Devon EX2 5DW, UK; College of Medicine and Health, University of Exeter, Exeter, Devon EX2 5DW, UK; GlaxoSmithKline, Boston, MA 02140, USA; College of Medicine and Health, University of Exeter, Exeter, Devon EX2 5DW, UK

## Abstract

Prolyl hydroxylase (PHD) inhibitors are in clinical development for anaemia in chronic kidney disease. Epidemiological studies have reported conflicting results regarding safety of long-term therapeutic haemoglobin (Hgb) rises through PHD inhibition on risk of cardiovascular disease. Genetic variation in genes encoding PHDs can be used as partial proxies to investigate the potential effects of long-term Hgb rises. We used Mendelian randomization to investigate the effect of long-term Hgb level rises through genetically proxied PHD inhibition on coronary artery disease (CAD: 60 801 cases; 123 504 controls), myocardial infarction (MI: 42 561 cases; 123 504 controls) or stroke (40 585 cases; 406 111 controls). To further characterize long-term effects of Hgb level rises, we performed a phenome-wide association study (PheWAS) in up to 451 099 UK Biobank individuals. Genetically proxied therapeutic PHD inhibition, equivalent to a 1.00 g/dl increase in Hgb levels, was not associated (at *P* < 0.05) with increased odds of CAD; odd ratio (OR) [95% confidence intervals (CI)] = 1.06 (0.84, 1.35), MI [OR (95% CI) = 1.02 (0.79, 1.33)] or stroke [OR (95% CI) = 0.91 (0.66, 1.24)]. PheWAS revealed associations with blood related phenotypes consistent with *EGLN’s* role, relevant kidney- and liver-related biomarkers like estimated glomerular filtration rate and microalbuminuria, and non-alcoholic fatty liver disease (Bonferroni-adjusted *P* < 5.42E-05) but these were not clinically meaningful. These findings suggest that long-term alterations in Hgb through PHD inhibition are unlikely to substantially increase cardiovascular disease risk; using large disease genome-wide association study data, we could exclude ORs of 1.35 for cardiovascular risk with a 1.00 g/dl increase in Hgb.

## Introduction

Chronic kidney disease (CKD) is a long-term condition characterized by progressive loss of kidney function. CKD affects between 8 and 16% of the global population and is most common in older populations and people of African American or Hispanic ethnicity (1,2). CKD is a heterogeneous disease with a variety of causes and symptoms and is diagnosed in stages ranging from mild/no symptoms to end-stage kidney failure (3,4). As CKD worsens, the risk of developing additional complications, such as anaemia, increases (5). Anaemia is associated with increased progression of CKD alongside increased risk of adverse cardiovascular events, including heart disease and stroke, poorer quality of life and higher mortality and morbidity (6–8). Anaemia in CKD often has more than one cause, including lower erythropoietin (EPO) levels, inflammation, bleeding and reduced iron availability all resulting in a reduced number of healthy circulating erythrocytes (9). Current treatments for anaemia in CKD include parenteral injections of recombinant human EPO (rhEPO) or its analogues, and iron therapies, which attempt to increase erythrocyte production and development to correct the anaemia (10,11). Despite potential benefits of these treatments including improvement in quality of life and reduced need for blood transfusions, they have limitations (6). Limitations of oral iron include poor compliance owing to gastrointestinal adverse effects and prolonged treatments, while limitations of rhEPO or intravenous iron include inconvenient administration (by injection or infusion, respectively), pain at the injection site and risk of adverse effects such as hypersensitivity with intravenous iron or hypertension with rhEPO (12–14). RhEPO also has additional safety concerns such as increased risk of venous thromboembolism, stroke, myocardial infarction (MI), heart failure and death owing to the supraphysiological EPO levels leading to sudden and/or excessive haemoglobin (Hgb) levels (15–18). These safety concerns have led to ongoing efforts to develop novel treatments for anaemia in CKD.

Hypoxia-inducible factor (HIF)-prolyl hydroxylase (PHD) inhibitors (PHIs) have recently completed phase III clinical trials for treating anaemia in CKD (6,19–24). PHIs act at the transcriptional level of the hypoxic-response genes by inhibiting the PHD enzymes (PHD1-3) leading to an accumulation of HIF-α activating the hypoxic response pathway (25,26). Increased transcription of the hypoxic-response genes results in increased erythropoiesis and subsequent elevated circulating Hgb levels restoring tissue oxygen delivery and correcting the anaemia (27–29). By acting at the transcriptional level, PHIs maintain endogenous EPO levels within the physiological range preventing sudden and/or excessive Hgb level elevations potentially reducing the risk of cardiovascular events, thromboembolism and heart failure compared with current treatments (25). Phase II trials indicate that PHIs can produce dose-dependent changes in Hgb levels and maintain target Hgb levels with small increases in EPO levels in patients either receiving or not receiving dialysis treatment (30–32). Phase III trials show PHIs to be non-inferior compared with rhEPO in terms of cardiovascular safety and haematological efficacy supporting ongoing development (19–23,33).

Genetic studies can be used to support clinical trial data by providing additional evidence that drug targets are associated with the intended therapeutic indication and not associated with unintended and non-beneficial effects further characterizing the therapeutic profile (34–36). Several examples already corroborate the power of genetic studies in providing supporting evidence of drug safety (37–39). Mendelian randomization (MR) is one approach in which genetics can be used to help identify causal relationships between intended (e.g. higher biomarker levels or disease) and unintended drug effects (e.g. disease or unintended effects) (40,41). Genetic variants lying within or nearby the gene encoding the drug target, or associated with the drug’s intended effects, are used as unconfounded, unbiased proxies for pharmacological action, providing evidence of life-long exposure on risk of disease (42). Phenome-wide association studies (PheWAS) are another method by which genetics can help characterize on-target therapeutic profile. In these studies, a genetic variant, or combination of variants, associated with the intended drug effects is tested for associations with a wide range of phenotypes in large sample sizes, to identify potential unexpected effects that may have not been considered in clinical trials (43).

PHIs target the hypoxic pathway through inhibition of the PHD enzymes encoded by the *EGLN* genes (*EGLN1/2/*3). Studies of rare genetic variants and *in vivo* models provide some insight into the potential effects of targeting the *EGLN* pathways (44). Some studies have shown that rare loss-of-function variants lying in *EGLN1* give rise to polycythaemia (pathogenic erythrocyte numbers) and inappropriate EPO production which is potentially linked to cardiovascular risk (e.g. hypertension or thrombotic events) in patients carrying these variants (44). Additionally, mice lacking *EGLN1* show embryonic lethality owing to heart and placental defects (45,46). However, these studies are limited by the small number of patients studied and the differences between humans and mice. Common *EGLN* gene variants with modest effects can therefore provide insight into the potential long-term effect of therapeutically altering Hgb in the physiological range through PHD inhibition.

In this study, we used common genetic variants, lying within or near the *EGLN* genes, to partially mimic PHD inhibition and assess the associated risk of cardiovascular disease [CVD: defined here as coronary artery disease (CAD), MI and stroke] with lifelong exposure to circulating Hgb level elevations through genetically proxied therapeutic PHD inhibition or other potential effects of targeting the *EGLN* genes.

## Results

### Genetically proxied therapeutic PHD inhibition resulting in long-term higher circulating Hgb levels is not associated with cardiovascular risk

**Figure 1:**
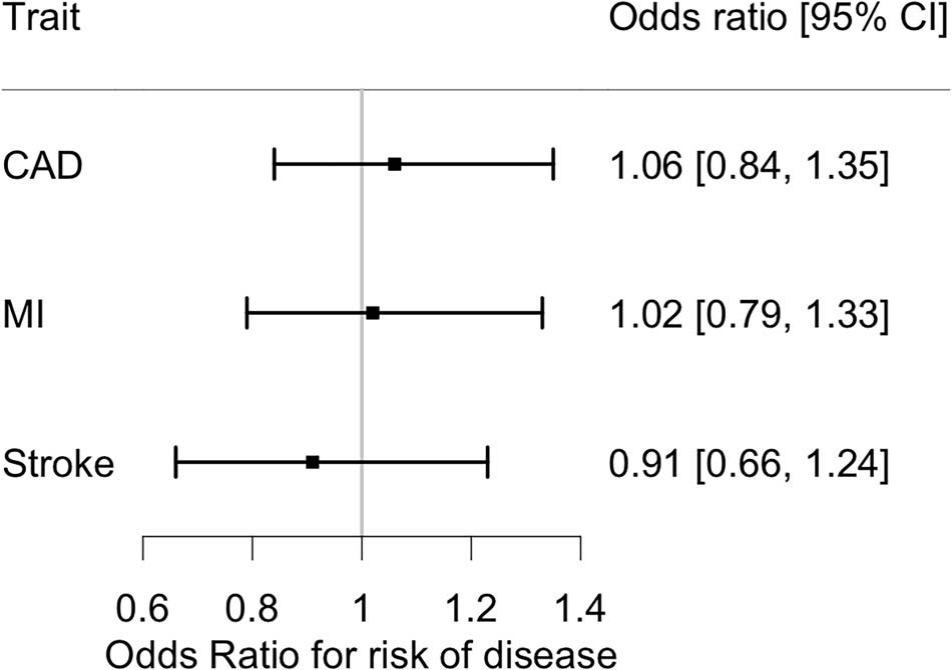
The effect of a 1-unit change in Hgb levels on risk of CVD in the general population as genetically proxied by therapeutic PHD inhibition. The genetic effects per 1 standard deviation increase in circulating Hgb levels were rescaled to the typical Hgb level in the general population by multiplying the effects by the 0.81 to represent a 1-unit increase in Hgb levels (e.g. going from 14.2 to 15.2 g/dL). Based on the upper bound of the estimate, we could exclude increased odds of 1.35 for CAD, increased odds of 1.33 for MI and increased odds of 1.24 for stroke with genetically proxied therapeutic PHD inhibition. Plot was produced using forestplot package in R.

To genetically proxy the effects of therapeutic PHD inhibition, we used eight Hgb-associated single nucleotide polymorphisms (SNPs) in three genes, *ELGN1, EGLN2* and *EGLN3,* encoding PHDs targeted by PHIs (Table 1). Using these variants as instruments in drug target two-sample MR, we found no evidence of a causal association with any of the three CVDs tested [inverse-variance weighted (IVW) estimates; CAD: OR (95% CI) = 1.02 (0.62, 1.66), *P* > 0.05; MI: OR (95% CI) = 1.03 (0.74, 1.42), *P* > 0.05; stroke: OR (95% CI) = 0.89 (0.61, 1.30), *P >* 0.05] (Supplementary Material, Fig. S1, Supplementary Material, Table S3). There was no evidence of pleiotropy or heterogeneity in the genetic instruments (Supplementary Material, Table S4). As common genetic variants tend to have subtle effects on phenotypes, it can be helpful to scale their effects to provide estimates in a more physiologically relevant range (47–49). We therefore present results of the estimated effect of a 1 g/dl increase in Hgb on CVD outcomes, based on genetic instrumentation of PHD inhibition (Supplementary Material, Table S5). We found no evidence (at *P* < 0.05) for increased odds of CAD [OR (95% CI) = 1.06 (0.84, 1.35)], MI [OR (95% CI) = 1.02 (0.79, 1.33)] or stroke [OR (95% CI) = 0.91 (0.66, 1.24)] for a 1-unit increase in Hgb level in the physiological range (e.g. from 14.2 to 15.2 g/dl) through genetically proxied PHD inhibition (Fig. 1, Supplementary Material, Table S5). Based on the upper confidence intervals, we could statistically exclude increased odds of 1.35, 1.33 and 1.24 for CAD, MI or stroke, respectively (Fig. 1, Supplementary Material, Table S5).

**Table 1:** Association between the eight EGLN specific genetic variants and circulating Hgb levels

Gene	Ref Seq	RSID	Chr	Pos	Type of variant	Evidence	Ref allele	Effect allele	Effect allele freq	Effect estimate for effect allele	SE	*P*-value	N
*EGLN1*	NM_022051.3	rs999010	1	231 495 316	Downstream gene variant	eQTL	A	G	0.63	0.03	0.002	1.17E-36	408 122
*EGLN1*	NM_022051.3	rs61835223	1	231 562 228	Upstream gene variant	Nearest gene & eQTL	A	G	0.02	0.12	0.008	2.55E-55	408 122
*EGLN2*	NM_080732.4	rs73047068	19	41 297 106	Intron variant	Nearest gene	C	G	0.84	0.02	0.003	2.25E-10	408 122
*EGLN2*	NM_080732.4	rs192191487	19	41 305 065	Intron variant	Nearest gene	G	A	0.02	0.08	0.009	2.84E-18	408 122
*EGLN2*	NM_080732.4	rs184088518	19	41 305 138	5 prime UTR variant	Nearest gene	T	G	0.98	0.12	0.007	3.65E-60	408 122
*EGLN2*	NM_080732.4	rs61750953	19	41 306 650	Missense variant	Coding	T	C	0.99	0.10	0.009	2.74E-28	408 122
*EGLN3*	NM_022073.4	rs797343	14	34 646 269	Intron variant	eQTL	C	T	0.68	0.02	0.002	4.43E-19	408 122
*EGLN3*	NM_022073.4	rs12897414	14	34 724 550	Intron variant	Most severe coding sequence consequence based on VEP	T	C	0.38	0.01	0.002	4.12E-11	408 122

Effect estimates are aligned to the Hgb-increasing allele and were obtained from the recently published GWAS on Hgb levels (51). OpenTargets (https://genetics.opentargets.org/variant) was used to determine the best line of evidence mapping the variants to the EGLN gene. UTR = untranslated region, eQTL = expression quantitative trait loci, Ref = Reference, SE = standard error, N = number.

### The *EGLN*-specific Hgb genetic risk score is associated with relevant erythrocyte traits and biomarkers related to kidney function, indicating the *EGLN* SNPs are likely specific instruments to mimic therapeutic PHD inhibition

**Figure 2:**
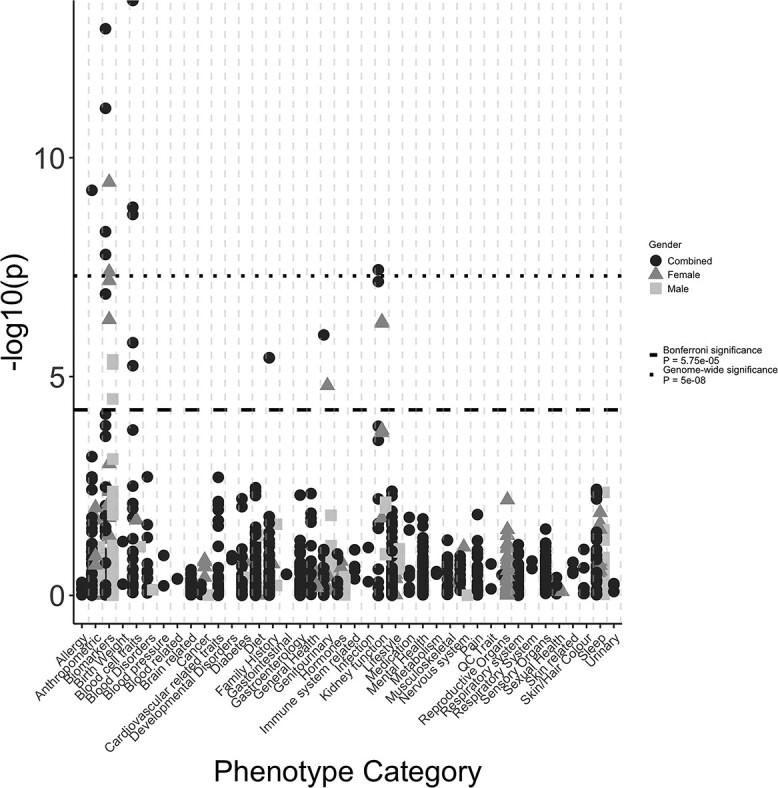
PheWAS of the *EGLN*-specific Hgb GRS with 923 traits in up to 451 099 unrelated, European UKB individuals. The Hgb GRS is most strongly associated with erythrocyte traits indicating that the *EGLN*-specific variants are likely acting through the hypoxic pathway and are valid and specific proxies for therapeutic PHD inhibition. We also found strong associations with relevant biomarkers. Long-term genetically mediated Hgb levels are unlikely to increase risk of additional comorbidities or unintended effects. The traits have been clustered into relevant categories.

To determine the specificity of the *EGLN*-specific SNPs as instruments for Hgb levels, we generated a weighted Hgb genetic risk score (GRS) consisting of the eight SNPs. We then used this GRS to perform a PheWAS on 923 traits in up to 451 099 unrelated European UK Biobank (UKB) individuals regardless of CKD status. The weighted Hgb GRS was associated with 0.05 standard deviations (SD) (SE = 0.002, *P* = 8 × 10^−168^) higher circulating Hgb levels, equivalent to a per allele 0.062-unit increase in Hgb in the general population (Supplementary Material, Table S6). We found the *EGLN*-specific Hgb GRS was most strongly associated with erythrocyte phenotypes including red blood cell count [beta (SE) = 0.05 (0.002), *P* = 5.00 × 10^−120^], haematocrit percentage [beta (SE) = 0.05 (0.002), *P* = 2.00 × 10^−168^], reticulocyte count [beta (SE) = 0.01 (0.002), *P* = 1.98 × 10^−09^], platelet crit [beta (SE) = −0.01 (0.002), *P* = 1.68 × 10^−06^] and platelet count [beta (SE) = −0.01 (0.002), *P* = 5.64 × 10^−06^] (Fig. 2, Supplementary Material, Table S6). We also found associations between the *EGLN*-specific Hgb GRS and traits related to kidney function, including creatinine-based estimated glomerular filtration rate (eGFR) [beta (SE) = −0.01 (0.002), *P* = 3.63 × 10^−08^] and microalbumin [beta (SE) = −0.01 (0.002), *P* = 4.88 × 10^−09^] (Fig. 2, Supplementary Material, Table S6) and liver function related traits, such as bilirubin [total bilirubin: beta (SE) = 0.02 (0.002), *P* = 7.40 × 10^−12^], a biomarker indicative of erythrocyte disorders (50). Despite being statistically significant, these associations were not clinically significant (equivalent to a 2.22, 0.02 and 1.08-unit change in eGFR, microalbumin and total bilirubin per 1 g/dl higher Hgb, respectively). Stronger associations, passing the Bonferroni *P*-value threshold (*P* < 5.42 × 10^−05^), were found in women compared with men for bilirubin, microalbumin, creatinine and eGFR, although the direction and magnitude of effects remained consistent (Fig. 2, Supplementary Material, Table S6).

### Long-term elevated circulating Hgb levels through genetically proxied therapeutic PHD inhibition is unlikely to severely increase risk of other comorbidities

To identify potential additional unintended, non-beneficial effects associated with long-term increasing Hgb levels through genetically proxied therapeutic PHD inhibition, we tested the *EGLN-*specific Hgb GRS for association with 923 traits in up to 451 099 unrelated European UKB individuals regardless of CKD status. We found evidence for an association with reduced sitting-to-standing height ratio [beta (SE) = −0.01 (0.002), *P* = 5.54 × 10^−10^], and increased risk of non-alcoholic fatty liver disease (NAFLD) fibrosis score [beta (SE) = 0.01 (0.002), *P* = 1.12 × 10^−06^] with higher genetically mediated Hgb levels (Fig. 2, Supplementary Material, Table S6). However, this association was not determined clinically significant (equivalent to a 0.18 change in NAFLD for a 1 g/dl increase in Hgb levels). We also observed an association with family history of diabetes in siblings [OR (95% CI): 1.04 (1.02, 1.06), *P* = 3.71 × 10^−06^] but this was not consistent with the result of type 2 diabetes risk in participants [OR (95% CI): 0.99 (0.97, 1.03), *P* = 0.998]. Overall, these results indicate that long-term higher circulating Hgb levels through therapeutic inhibition of PHDs are unlikely to confer an increased risk of any secondary conditions at clinical levels of significance (Fig. 2, Supplementary Material, Table S6).

### Secondary analysis focusing on overall genetically proxied long-term rises in Hgb levels, not necessarily through PHD inhibition, showed no increase in cardiovascular risk

To understand the effects of general long-term rises in Hgb levels on cardiovascular risk, which is not specific to therapeutic PHD inhibition, we performed two-sample MR using 515 Hgb-associated SNPs as instruments. We selected 515 conditionally independent genetic variants associated (at *P* < 5 × 10^−09^) with circulating Hgb levels from the most recent, publicly available genome-wide association study (GWAS) on blood cell traits (51) (Supplementary Material, Table S1). Summary statistics for 409, 407 and 410 of the Hgb-associated variants were available in the publicly available GWAS of the three CVD of interest, CAD, MI or stroke, respectively (52,53) (Supplementary Material, Table S2). We found no evidence (at *P* < 0.05) that a 1-unit increase in genetically mediated Hgb levels in a physiological range leads to an increased risk of stroke [OR (95% CI): 1.04 (1.00, 1.08), *P* = 0.08] or CAD [OR (95% CI): 1.05 (1.00, 1.11), *P* = 0.07] in the general population (Supplementary Material, Fig. S2, Supplementary Material, Table S7). We found nominal evidence for an association between a 1-unit increase in genetically mediated Hgb levels and increased risk of MI [OR (95% CI): 1.08 (1.02, 1.14), *P* = 0.01] (Supplementary Material, Fig. S2, Supplementary Material, Table S7) but there was strong evidence of pleiotropy and heterogeneity for both the CAD and MI estimates (Egger intercept *P*-value: CAD = 1.68 × 10^−05^, MI = 3.80 × 10^−05^, heterogeneity *P*-value IVW: CAD = 2.11 × 10^−45^, MI = 2.81 × 10^−34^, from Supplementary Material, Table S8).

### Steiger filtering strengthens results of the overall genetically proxied long-term rises in Hgb levels

To reduce the level of pleiotropy and heterogeneity when using the 515 Hgb-associated variants, we performed Steiger filtering (54). By applying a Steiger filtering false discovery rate threshold of 0.05 to limit the selected variants to those with a greater effect on the exposure than the outcome, the number of variants used to assess the relationship between higher Hgb levels and risk of CAD, MI or stroke reduced by 107, 156 and 114, respectively (Supplementary Material, Table S9). After applying Steiger filtering, the direction of effect of the causal estimates between MR methods was more consistent, and the amount of heterogeneity and pleiotropy decreased but the confidence intervals were wider (Supplementary Material, Fig. S3, Supplementary Material, Tables S10 and S11). Using these filtered Hgb-associated variants, we again found no evidence (at *P* < 0.05) of a causal association between higher genetically mediated circulating Hgb levels and increased risk of CAD [OR (95% CI): 1.01 (0.96, 1.07), *P* = 0.62], MI [OR (95% CI): 1.03 (0.97, 1.10), *P* = 0.34] or stroke [OR (95% CI): 1.05 (0.99, 1.10), *P* = 0.05] (Supplementary Material, Fig. S3, Supplementary Material, Table S10).

## Discussion

Previous research has shown how human genetics can be used to further characterize therapeutic profiles and help anticipate the risk of unintended effects. PHIs have recently completed phase III clinical trials to treat anaemia of CKD (19–24,55,56). These phase III trials have shown non-inferiority for haematologic efficacy, and some non-inferiority for cardiovascular safety, with PHI treatment compared with rhEPO (19–21). We used human genetic variants associated with circulating Hgb levels as genetic proxies for the pharmaceutical effect of PHIs and investigate lifelong exposure to higher circulating Hgb levels. We provide genetic evidence to support cardiovascular safety of PHIs and further inform on potential risk of other effects of therapeutic PHD inhibition which may not be tested in clinical trials. We used a drug target-specific (*EGLN1/2/3)* Hgb genetic instrument to partially mimic the direct effects of therapeutic PHD inhibition through PHI treatment and found no evidence of a causal association between higher Hgb levels and increased cardiovascular risk (Supplementary Material, Fig. S1). We rescaled our genetic estimates obtained using the *EGLN*-specific instrument to the Hgb levels typically found in the general population to obtain a more relevant effect estimate on the physiological scale (Fig. 1). Based on the upper bound, we could exclude a 1.35, 1.33 and 1.24 increased odds of CAD, MI or stroke, respectively, with long-term therapeutic rises in Hgb levels of 1 g/dl (Fig. 1). As all PHIs work through the same mechanisms (i.e. inhibition of PHDs), these results are likely supportive of all PHIs. Any differences seen between PHI compounds would be likely related to the biochemical and physical properties of the compounds and way the treatment is used particularly regarding dosing.

Our results provide genetic support of the findings from clinical trials in that PHIs are non-inferior for CVD than rhEPO for treating anaemia in CKD patients. These findings could also be used to support development of treatments for other diseases which act by increasing Hgb levels through the hypoxic pathway, highlighting the translational ability of these types of genetic studies to help predict the risk of potential unintended effects or benefits of any treatment for any disease undergoing clinical development (35). However, it is important to consider the validity of the genetic instrument used in terms of how well the SNP mimics the pharmacological action of the drug and the strength of the variant as an instrument (41). MR analysis makes several assumptions and violation of these assumptions can lead to bias in the causal estimates (57). Here, we used both a specific set of SNPs in or near the *EGLN* genes and a wider set of variants associated with Hgb. When using all the Hgb-associated SNPs to assess the causal relationship between higher Hgb and risk of CVD, we found evidence of pleiotropy (Egger-intercept *P*-value < 0.05, Supplementary Material, Table S8) (58) but showed that limiting the variants to those with larger effects on the exposure compared with the outcome (through Steiger filtering) reduced the pleiotropy and heterogeneity increasing power to detect causality in the true direction (Supplementary Material, Figs S2 and S3, Supplementary Material, Tables S10 and S11) (59).

As with all uses of common variants as genetic proxies of drug interventions, there are limitations. First, genetic variants tend to represent subtle lifelong changes rather than the more acute and stronger changes from therapies (60,61). Second, and most importantly, the genetic effects are based on estimates of Hgb alterations in the general population, regardless of CKD status, whereas PHI therapies are given only to anaemic patients with Hgb levels towards the lower end of the range. CKD patients are likely to have variable biomarker levels at baseline which could alter the causal estimates and the presence of other underlying conditions which could alter the way they respond to therapeutic PHD inhibition than that estimated by the genetic association (62,63). The majority of genetic analyses assume linearity, which is not always the case, particularly in relation to PHI treatment which is titrated at individual patient level to achieve a target Hgb level meaning patients will have different baseline Hgb levels and subsequent increases in Hgb levels from baseline (63). Third, it is often difficult to represent the efficacious physiologically relevant state or representative cellular concentration of a drug target using genetics as the genetic estimate often reflects circulating levels (64). Despite rescaling genetic effects to the physiological relevant effect to try and overcome some of these limitations, we remain limited in our ability to transfer these findings to the target patient population. As more extensive genetic studies become available, particularly in disease-relevant populations, our power to detect associations and our ability to perform stratified analyses at different baseline levels will improve (42,51,63,65,66). To provide additional evidence that the variants were specific and valid proxies for therapeutic PHD inhibition and further insight into the potential effects of PHD inhibition, we performed a PheWAS. PheWAS has potential for improving or validating our understanding of biological mechanism, identifying additional indications with potential for repurposing, or indicating potential unwanted effects through associations with other conditions other than the primary indication (61,67,68). Through our PheWAS, we found the weighted Hgb GRS to be most strongly associated with relevant erythrocyte phenotypes, such as platelet count and red blood count, indicating that these variants are strong, valid genetic instruments as they appear to influence circulating Hgb levels through altered erythropoiesis, the downstream effect of PHD inhibition (Fig. 2, Supplementary Material, Table S6). We also found additional associations with relevant kidney and liver function related biomarkers, such as eGFR, microalbuminuria and bilirubin. Although these did not reach clinical significance, they further indicate that these instruments are likely acting through the hypoxic pathway in relevant tissue types (where EPO is predominantly produced) (Fig. 2, Supplementary Material, Table S6) (29,69). However, the direction of effect of higher genetically determined Hgb via the *EGLN* genes on these biomarkers appears counterintuitive; higher Hgb levels are associated with lower eGFR indicative of worse kidney function but with lower microalbuminuria which is a marker of healthier kidneys. Higher Hgb levels are also associated with increased bilirubin, which may be indicative of haemolysis leading to lower Hgb, not higher Hgb. Sex-specific PheWAS revealed stronger associations (based on *P*-values) between higher Hgb levels and several of the biomarkers, such as bilirubin, creatinine and eGFR, in women compared with men which suggests that higher Hgb levels have a greater effect in women (Fig. 2, Supplementary Material, Table S6). Women, in general, have lower Hgb levels than men so increasing Hgb in women is expected to have a larger effect than in men who already have higher Hgb baseline levels (70). Women are often underrepresented in clinical trials, so our study, using genetics as proxies for drug effects, is a useful additional way of increasing relevance to a wider range of patients (71–73).

When looking for associations with potential secondary diseases or unintended effects, we found evidence for an association between the *EGLN*-specific Hgb GRS and risk of NAFLD although this association did not reach clinical levels of significance (Fig. 2, Supplementary Material, Table S6). The *EGLN* genes are known to play a role in glucose metabolism through activation of HIF-2a, and this likely explains the association found between the *EGLN* SNPs and NAFLD (from metabolic syndrome) (74–76). NAFLD is also prevalent in CKD patients and is a clinical marker of poor response to EPO treatments and could therefore be used to determine response to therapeutic PHD inhibition (77). As these investigations were performed on the general population, it is unclear whether there is some sort of feedback mechanism or confounding impacting these findings and whether inference can be made to a CKD population. It would, therefore, be worth investigation in a CKD-specific population. Furthermore, PheWAS was performed in UKB individuals, within whom the instruments were discovered. These overlapping samples may bias our results in the direction of the observational data (78).

In conclusion, our results suggest that general long-term elevated circulating Hgb levels through genetically proxied therapeutic PHD inhibition do not increase risk of CVD or additional complications. We have identified relevant genetic markers for testing the pharmaceutical effects of therapeutic PHD inhibition which could potentially inform further research using patient level clinical data from phase III trials. We show additional evidence of how human genetics can be used to partially mimic pharmacological action and provide additional insight, alongside clinical trial data, into the long-term therapeutic effects.

## Materials and Methods

### Selection of Hgb-associated genetic variants

Using the most recent published GWAS of Hgb, we extracted the publicly available summary association statistics for 515 conditionally independent SNPs [minor allele frequency (MAF) > 1%] associated with Hgb levels at *P* < 5 × 10^−09^ as identified by Vuckovic *et al*. (51). We (51) aligned effect sizes to the Hgb-increasing allele (Supplementary Material, Table S1). These statistics were based on the 408 112 Europeans studied in UKB.

### Selection of drug target-specific Hgb-associated SNPs

From the list of 515 conditionally independent Hgb-associated genetic variants identified by Vuckovic *et al.* (51), we selected eight SNPs annotated to three *EGLN* genes [*EGLN1* (ENSG00000135766), *EGLN2* (ENSG00000269858), *EGLN3* (ENSG00000129521)] encoding PHI drug targets (PHD1-3). A gene symbol was provided for the Hgb-associated SNPs by Vuckovic *et al*. (51) based on the variant effect predictor (VEP) annotation tool (79), assigning the gene symbol(s) for the most serious predicted consequence. OpenTargets (https://genetics.opentargets.org/variant) was used to determine the best line of evidence mapping the variant to each *EGLN* gene. For the variants which had eQTL evidence, the association between the variant and corresponding *EGLN* gene expression was found in the blood using the eQTLGen data set. One of the variants lies within an exon of *EGLN2* and disrupts the coding sequence [rs61750953; serine (TCG) > Leucine (TTG); Ref Seq NM_080732.4], one lies within the 5′ UTR of *EGLN2* (rs184088518 G>T; Ref Seq NM_080732.4) and the others are in non-coding sequence near the *EGLN* genes (Table 1). We extracted summary statistics for the association between these eight *EGLN-*specific SNPs and circulating Hgb levels from Vuckovic *et al*. (51).

### Definition of CVD

We selected three CVDs—stroke, MI or CAD—given their relevance to PHIs and the availability of GWAS data from very large samples. We obtained summary association statistics for the 515 Hgb-associated SNPs on CAD, MI or stroke from recently published, publicly available GWAS in European individuals which did not include UKB individuals to ensure estimates came from independent cohorts increasing statistical power and reducing risk of ‘winner’s curse’ (whereby the true causal estimate can be underestimated) (80). For MI and CAD, we used the GWAS performed by Nikpay *et al*. (52) in 42 561 and 60 801 cases respectively and 123 504 controls (Supplementary Material, Table S2). CAD was defined by a record of MI, acute coronary syndrome, chronic stable angina or coronary stenosis > 50% (based on coronary angiographic evidence) obtained from patient and death registers (see Nikpay *et al.* (52) for additional details). For stroke, we used the GWAS performed by Malik *et al*. (53) in 40 585 cases and 406 111 controls (Supplementary Material, Table S2). Stroke was defined as ischaemic stroke or intracerebral haemorrhage based on clinical and imaging criteria (53). Subarachnoid haemorrhages were excluded (53). We did not look for proxies for the SNP (rs192191487) which was missing in the stroke GWAS (53).

### Two-sample MR analysis

We performed two-sample MR analysis using the MRBase package (81) implemented in R (82). Palindromic SNPs with intermediate allele frequencies were removed. We first performed drug target two-sample MR using the eight drug target-specific Hgb-associated *EGLN* SNPs as instruments and then performed secondary analysis using the 515 Hgb-associated SNPs. Five two-sample MR methods were performed: IVW; MR Egger (58); weighted median (83); weighted mode (83); simple mode (84). We have presented the IVW approach as our main analysis method, with the latter four representing sensitivity analyses to account for unidentified pleiotropy which may bias our results. IVW assumes there is no horizontal pleiotropy (where genetic variants influence the outcome independently of the exposure) and that the SNP-exposure association is not correlated with the path from SNP outcome that is independent of the exposure (InSIDE assumption) (85,86). We tested for pleiotropic effects using the MR Egger intercept obtained through the ‘mr_pleiotropy_test’ function and for heterogeneity using the ‘mr_heterogeneity’ function (58). When there was evidence of pleiotropy (indicated by *P* < 0.05), we placed more weighting on the MR Egger estimate, which partially accounts for pleiotropic effects and provides unbiased estimates.

### Steiger filtering

To obtain the most specific Hgb genetic instrument, we performed Steiger filtering (54,59) using the MRBase package (81) in R (82) on the 515 Hgb-associated SNPs. Steiger filtering uses a statistical method to select those genetic variants which explain more variance in the exposure than the outcome [R^2^(exposure) > R^2^(outcome)] (59). We filtered the 515 Hgb-associated genetic variants to obtain a more specific instrument with primary effects on the exposure using Steiger_direction = true and Steiger *P*-value < 0.05. We repeated two-sample MR using this filtered Hgb-specific set of 288, 237 and 284 genetic variants to obtain a more reliable estimate of the relationship between long-term genetically proxied Hgb levels and cardiovascular risk. We did not perform Steiger filtering when performing MR using the *EGLN-*specific genetic instruments as these variants had stronger effects on Hgb than the heart disease traits and showed no evidence of pleiotropy or heterogeneity (Supplementary Material, Table S4).

### Comparison of effect estimates to typical Hgb levels in general population

To obtain a more representative, physiologically relevant effect, we scaled the genetic effect estimates on disease outcomes by a factor of 0.81 (1/1.23), where 1 is the desired unit change of Hgb in raw units (g/dl) and 1.23 is the standard deviation of Hgb in the UKB. This value provided an estimate of the genetically proxied odds of disease for a 1-unit increase in long-term circulating Hgb levels. A 1-unit increase in Hgb levels is the minimally clinically significant increase.

### PheWAS of an *EGLN*-specific GRS

To investigate the potential pleiotropic effects of the eight *EGLN-*specific SNPs or identify other potential effects downstream of Hgb through targeting the *EGLN* genes, we performed a PheWAS on 923 traits in up to 451 099 unrelated, European UKB individuals using a weighted GRS consisting of the eight *EGLN*-specific Hgb-associated SNPs. Traits were selected following the same approach as Frayling *et al*. (87). We extracted the dosages of the *EGLN-*specific SNPs from 437 573 unrelated European UKB individuals, as defined by principal component (PC) analysis (method details in (87)), with phenotypic and genotypic information. SNP genotype dosages were aligned to the number of Hgb-increasing alleles. We created the weighted GRS using the following equation:

$$ \mathrm{Weighted}\ \mathrm{Hgb}\ \mathrm{GRS}=\sum \mathrm{dosage}\times \mid \beta \left(\mathrm{Hgb}\right)\mid $$

To obtain all genotype–phenotype associations, regression analysis of the weighted GRS on 923 traits adjusting for age, sex, chip, centre and PCs 1–5 was performed. Continuous traits were inverse normalized prior to regression to account for skewed distributions. We stratified the traits by sex as well to investigate any sex-specific effects. We highlight associations reaching a Bonferroni-adjusted *P*-value < 5.42 × 10^−05^ (0.05/923). We converted effect estimates back to original units to determine whether statistically significant associations were clinically significant using the standard deviation of phenotypes in the UKB.

All analyses described before were decided a priori. All statistical analyses were performed using R version 3.6.1 or Stata version 16.1.

## Supplementary Material

SupplementaryTables_EGLN_HMG_ddac215

Supplementary_Figures_ddac215
