# Supplementary material for: Genetically proxied therapeutic prolyl-hydroxylase inhibition and cardiovascular risk
Source: Hum Mol Genet. 2022 Sep 1;32(3):496–505. doi: 10.1093/hmg/ddac215 (PMC9851745; doi:10.1093/hmg/ddac215)
Supplement: Supplementary_Figures_ddac215 [file supplementary_figures_ddac215.zip › Supplementary_Figures_ddac215.pdf]

Supplementary Figures

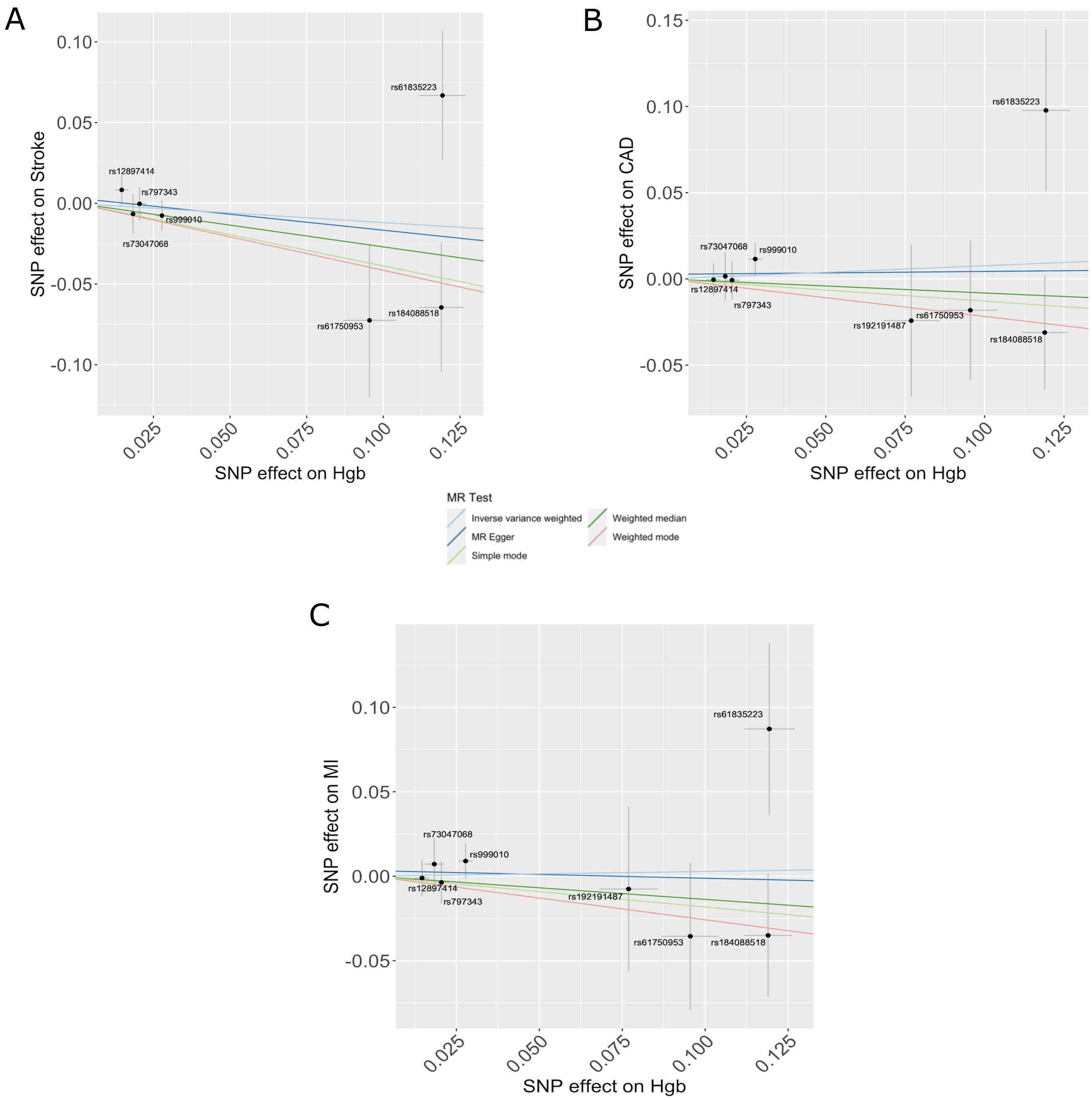

Supplementary Figure S1 Genetically proxied therapeutic PHD inhibition shows no evidence of adverse cardiovascular risk with long-term higher circulating Hgb levels (at $P < 0.05$).
Two sample Mendelian Randomisation was carried out using the eight *EGLN*-specific Hgb-associated variants to genetically mimic therapeutic PHD inhibition. The inverse-variance weighted method was the main analysis (light blue line) and the remaining four were used for sensitivity (MR Egger = dark blue line, Simple mode = light green line, Weighted median = dark green line, Weighted mode = red line). MR causal estimates were consistent across all methods, including sensitivity methods. There was no evidence of heterogeneity or pleiotropy. One of the eight EGLN-specific variants was missing from the stroke GWAS (rs192191487) hence why only seven instruments were included for determining the MR estimate between EPO and stroke. Plots were produced using the TwoSampleMR package in R. CAD = coronary artery disease, MI = myocardial infarction, Hgb = haemoglobin, SNP = single nucleotide polymorphism.

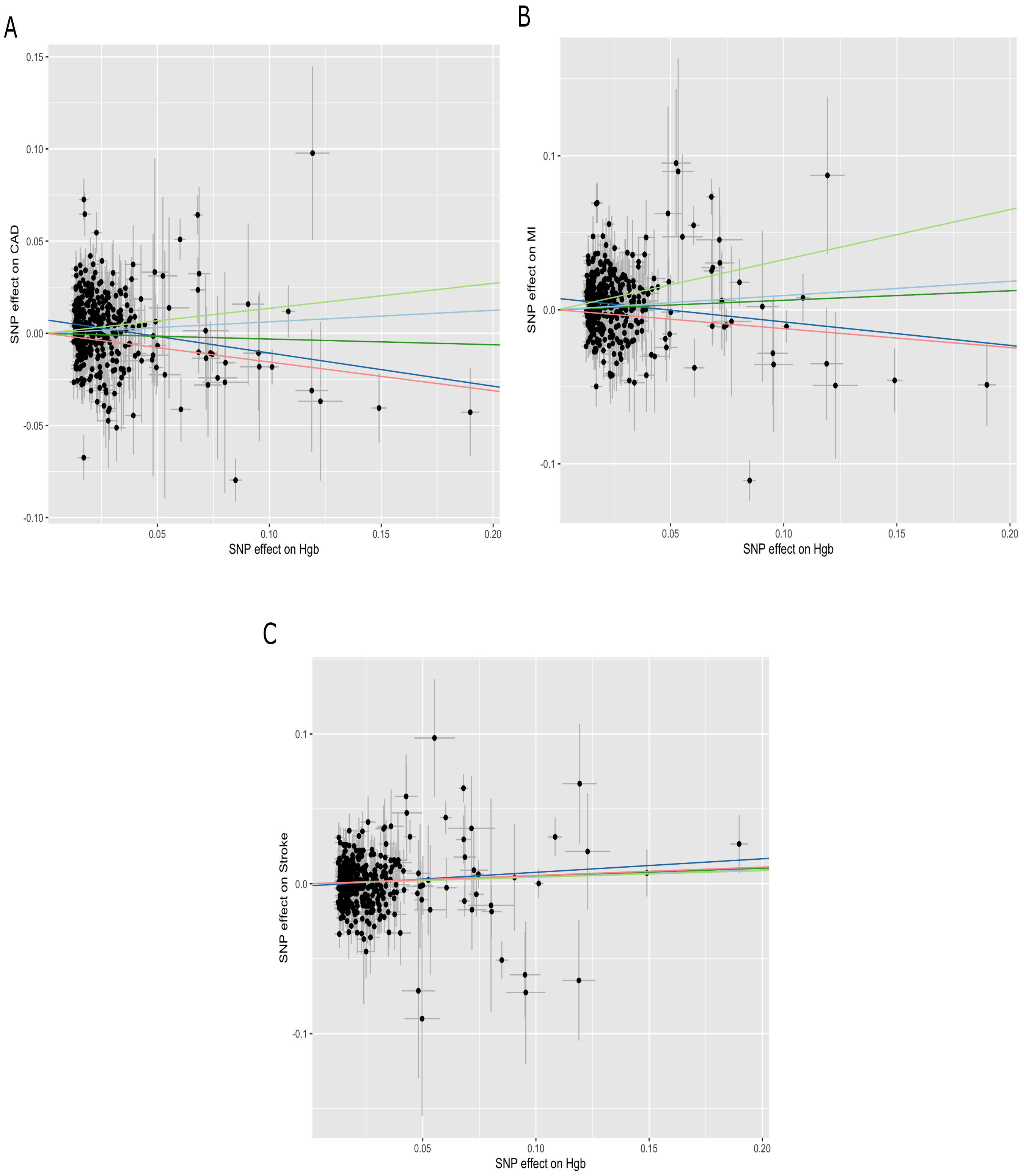

Supplementary Figure S2 MR estimates for the association between Hgb levels and cardiovascular risk using 515 Hgb-associated SNPs as genetic instruments before Steiger filtering.

a) The causal association between Hgb levels and risk of CAD. b) The causal association between Hgb levels and risk of MI. c) The causal association between Hgb levels and risk of stroke. There was presence of pleiotropy and heterogeneity in the instruments when testing the association between higher Hgb levels and CAD or MI and therefore more weighting was placed on the MR Egger estimate. The different colour lines represent the five different MR tests (light blue: inverse variance weighted, dark blue: MR Egger, light green: simple mode, dark green: weighted median, red: weighted mode). Plots were produced using the *TwoSampleMR* package in R.

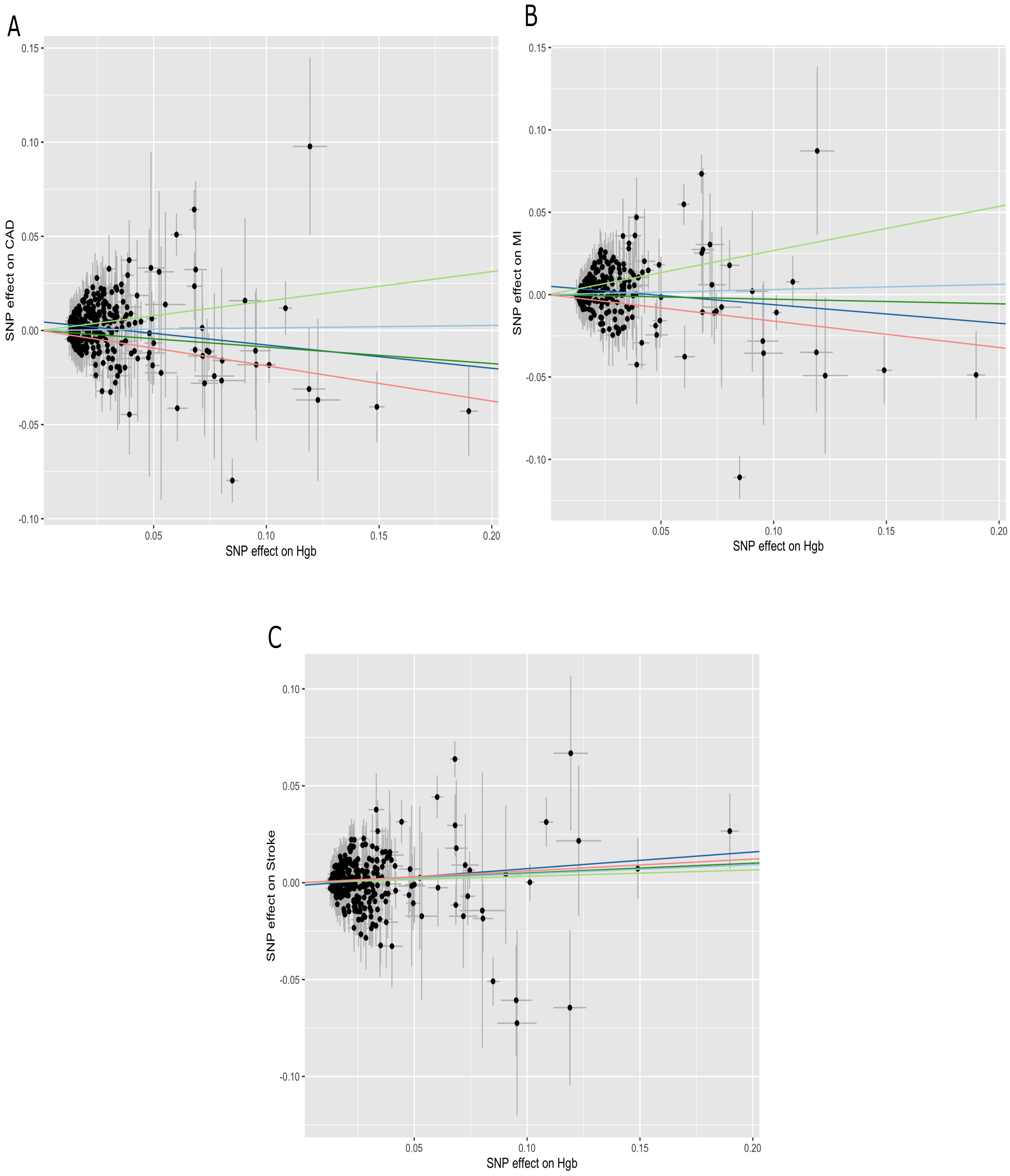

Supplementary Figure S3 MR estimates for the association between Hgb levels and cardiovascular risk using a more specific set of Hgb-associated SNPs as genetic instruments after Steiger filtering.

During Steiger filtering, we filtered for SNPs which explained higher variance in Hgb levels compared to the disease outcomes and passed a Steiger *P*-value threshold < 0.05. Estimates across the five methods became more consistent after Steiger filtering increasing reliability of the true causal estimate. a) The causal association between Hgb levels and risk of CAD. b) The causal association between Hgb levels and risk of MI. c) The causal association between Hgb levels and risk of stroke. The different colour lines represent the five different MR tests (light blue: inverse variance weighted, dark blue: MR Egger, light green: simple mode, dark green: weighted median, red: weighted mode). Plots were produced using the *TwoSampleMR* package in R.
